# Ethnic disparity in diagnosing asymptomatic bacterial vaginosis using machine learning

**DOI:** 10.1038/s41746-023-00953-1

**Published:** 2023-11-17

**Authors:** Cameron Celeste, Dion Ming, Justin Broce, Diandra P. Ojo, Emma Drobina, Adetola F. Louis-Jacques, Juan E. Gilbert, Ruogu Fang, Ivana K. Parker

**Affiliations:** 1 J. Crayton Pruitt Family Department of Biomedical Engineering, University of Florida, Gainesville, FL 32610 USA; 2 Department of Computer and Information Science and Engineering, University of Florida, Gainesville, FL 32611 USA; 3 Department of Obstetrics and Gynecology, College of Medicine, University of Florida, Gainesville, FL 32611 USA; 4 Department of Electrical and Computer Engineering, University of Florida, Gainesville, FL 32611 USA; 5 Department of Radiology, University of Florida, Gainesville, FL 32611 USA

**Keywords:** Machine learning, Microbiome, Bacterial pathogenesis

## Abstract

While machine learning (ML) has shown great promise in medical diagnostics, a major challenge is that ML models do not always perform equally well among ethnic groups. This is alarming for women’s health, as there are already existing health disparities that vary by ethnicity. Bacterial Vaginosis (BV) is a common vaginal syndrome among women of reproductive age and has clear diagnostic differences among ethnic groups. Here, we investigate the ability of four ML algorithms to diagnose BV. We determine the fairness in the prediction of asymptomatic BV using 16S rRNA sequencing data from Asian, Black, Hispanic, and white women. General purpose ML model performances vary based on ethnicity. When evaluating the metric of false positive or false negative rate, we find that models perform least effectively for Hispanic and Asian women. Models generally have the highest performance for white women and the lowest for Asian women. These findings demonstrate a need for improved methodologies to increase model fairness for predicting BV.

## Introduction

Bacterial vaginosis (BV) is a common vaginal syndrome among women of reproductive age and is associated with a multitude of adverse complications including the risk of preterm labor^1^, pelvic inflammatory disease^2–4^, STIs^5,6^, and HIV infection^7,8^. The global prevalence for BV is high, ranging from 21% to 29%, with disproportionately higher diagnoses in Black and Hispanic women^9,10^. BV is characterized by a non-optimal vaginal microbiome, with a shift away from a low-diversity profile dominated by lactobacilli to a high-diversity profile with an increase in anaerobic organisms (i.e., *Gardnerella vaginalis* and *Prevotella bivia*). Syndromic BV is characterized by vaginal discomfort, malodor, increased vaginal pH, and itching and is traditionally diagnosed using Amsel’s criteria and/or Nugent scoring^11,12^. However, a substantial number of women with BV are asymptomatic^13,14^ with an absence of vaginal discharge or odor, which causes difficulty in diagnosis and treatment^15^.

Recent advancements in high-throughput sequencing technologies highlight individual complexity and allow for the characterization of bacterial communities present within the vaginal microbiome^1,16^. Community State Types (CSTs) have emerged to further classify the vaginal microbiome with profiles being dominated by different *Lactobacilli* spp (group I, II, III, V) or without *Lactobacillus* dominance (group IV)^16^. CSTs provide structure to categorize the complexity of microbiome differences seen between individuals and identify trends among different ethnic groups. Black/African American women tend to have more diverse vaginal microbiomes than women of European descent even when healthy^17^. Indeed, the relationship between vaginal communities and BV are emerging to reveal the role of unique microbial combinations in disease progression^18,19^; the complexity of these interactions necessitate more comprehensive methods of analysis.

Artificial intelligence (AI) and machine learning (ML) can play a critical role in healthcare to improve patient outcomes and healthcare delivery. These tools can analyze vast amounts of medical data to identify patterns, make predictions, and offer personalized treatment plans that are tailored to an individual’s unique health profile. However, to ensure that AI and ML are effective and safe, they must be fair and unbiased. Prior research shows that ML models do not always perform equally well among ethnic groups due to bias or systematic errors in decision-making processes that can arise from various sources, including data collection, algorithm design, and human interpretation^20,21^. Furthermore, ML models can learn and replicate patterns of bias present in the training data, resulting in unfair or discriminatory outcomes^22,23^. Fair AI ensures that the algorithms are not influenced by factors such as ethnicity, gender, or socioeconomic status, which can further exasperate health disparities^24–27^.

In recent years, AI and ML have been used to analyze data for BV, (e.g., clinical symptoms, patient demographics, and microbiome profiles) and have identified factors that contribute to adverse health outcomes^24^. However, there is still an unmet need to determine how AI can be used to predict BV with fairness and to characterize unique features associated with BV that may vary based on ethnicity. These ethnicity-specific features provide insight into microbes that are important to consider in BV diagnosis and treatment. Within this context, ML studies can also identify common characteristics important for diagnosis, regardless of ethnicity.

ML is a useful tool to better understand these relationships and to leverage sequencing (seq) technologies to aid in BV treatment and diagnosis. For example, ML algorithms have been used as classifiers for 16S rRNA seq data to diagnose BV^28–30^ and delineate potential diagnostic features. However, these studies have not considered the fairness of predictions nor evaluated how metrics of model performance (e.g., balanced accuracy; false positive rate, FPR; false negative rate, FNR) vary among ethnic groups.

In this study, we determine the fairness in prediction of asymptomatic BV using 16S rRNA seq data from a diverse cohort of women. We compare several supervised ML models to assess model performance for each ethnicity and identify features that improve accuracy of prediction for each group (Asian, white, Black, Hispanic). The significance of evaluating fairness in AI/ML is evident as inaccuracies in diagnosis further exacerbate health disparities among vulnerable groups. The vaginal microbiome has been shown to vary based on ethnicity^17^, and ML tools provide insights into patient-specific strategies to consider in the development of improved diagnostics and therapeutics.

## Results

### Model performance varies between ethnicities

The dataset used in this study was previously described^16^. Vaginal species composition was characterized in vaginal fluid of 394 women by pyrosequencing of barcoded 16S rRNA genes at the V1-V2 region. Included in the dataset are the women’s ethnicities, the pH values of the vaginal swabs, and Nugent scores. Ethnicity classification terms include white, Black, Asian, and Hispanic. These terms were set by Ravel et al. and were self-identified by each patient. For this study, a Nugent score of 7 or greater is identified as BV positive^11^, otherwise the patient is identified as BV negative. Using this threshold, BV-negative patients are comprised of: 83 Asian, 62 Black, 65 Hispanic, and 87 white. For BV-positive patients this dataset includes: 13 Asian, 42 Black, 32 Hispanic, and 10 white.

The models used in this study were assessed using balanced accuracy, average precision, which is a calculation of the area under the precision-recall curve (AUPRC), false negative rate, and false positive rate.

**Fig. 1:**
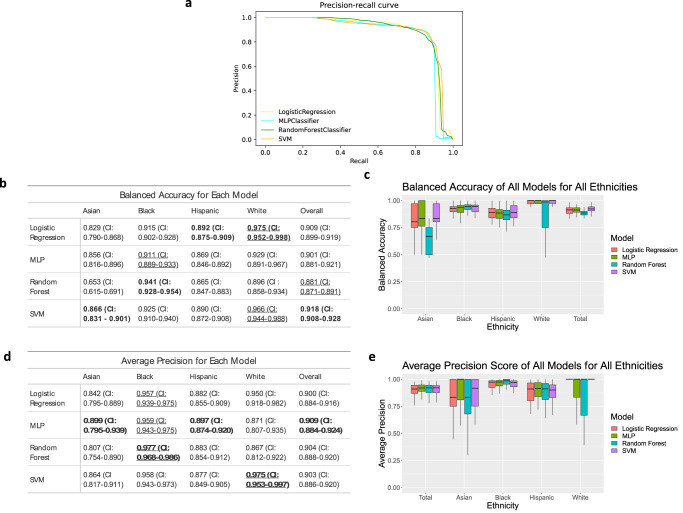
The testing of multiple machine learning models against specific ethnicities in the data set. Data was collected using 10 runs of 5-fold cross-validation. **a** Precision-recall curves for each model. **b**, **d** The balanced accuracy (**b**) and average precision (**d**) with 95% confidence interval of each model as an average of the 50 runs. Bold means the highest performance among methods (columns), and underline means the highest performance among ethnicities (rows). **c**, **e** Boxplots showing the median, upper quartile and lower quartile of the balanced accuracy (**c**) and average precision (**e**). Outliers are excluded. Values for boxplots are available in Supplementary Tables 1 and 2.

Figure 1 shows the balanced accuracy and average precision of four machine learning models (Logistic Regression (LR), Random Forest (RF), Support Vector Machine (SVM), Multi-layer Perceptron (MLP) classifiers) trained on the dataset. The AUPRCs for all four models (Fig. 1a) show that the general performance of each model is comparable. Balanced accuracy and average precision for these models vary from 0.881 and 0.918 (Fig. 1b) and 0.9 and 0.909 (Fig. 1d), respectively.

Evaluating balanced accuracy and average precision by ethnicity shows that the performance of these models varies between ethnicities. Using this dataset, balanced accuracies of the afore-mentioned models are highest for white women (.896-.975), followed by Black women (0.911–0.941), and Hispanic women (0.865–0.892). The models perform the least effectively for Asian women (0.653–0.866). The average precision (AP) follows a slightly different trend (Fig. 1d), with the highest performance for Black women (0.957–0.977), followed by white (0.867–0.975), Hispanic (0.877–0.897), and Asian (0.807–0.899) women, respectively. When evaluating how different classifiers performed, Random Forest performs best for Black women for both balanced accuracy and average precision; conversely, Random Forest tends to be the least accurate model when evaluated individually for white, Hispanic, and Asian patients. For all other models (LR, SVM, MLP), average precision and balanced accuracy are variable among ethnic groups.

**Fig. 2:**
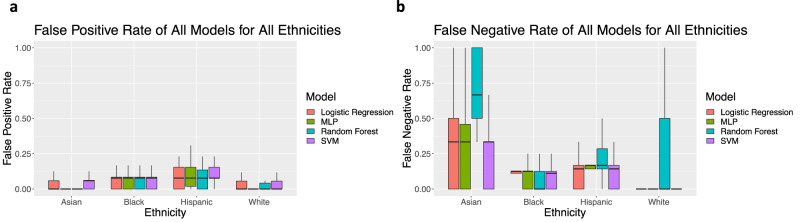
The outcomes by ethnicity from 10 runs of 5-fold cross-validation for multiple machine learning models and feature testing methods. **a** The false positive rate of each model by ethnicity as an average of the 50 runs. **b** The false negative rate of each model by ethnicity as an average of the 50 runs. Outliers are excluded. Values for boxplots are available in Supplementary Tables 3 and 4.

When evaluating false positive rates (FPR) by ethnicity, models tend to perform better for Asian and white women than Hispanic and Black women (Fig. 2a). When evaluating false negative rates (FNR) by ethnicity, the models tend to perform better for Black and white women than Hispanic and Asian women (Fig. 2b). When testing on general training sets, the SVM model has the best overall performance in balanced accuracy. For this reason, results of later experiments are only shown for the SVM model.

### Training the models on subsets of ethnicities yields limited changes in performance

**Fig. 3:**
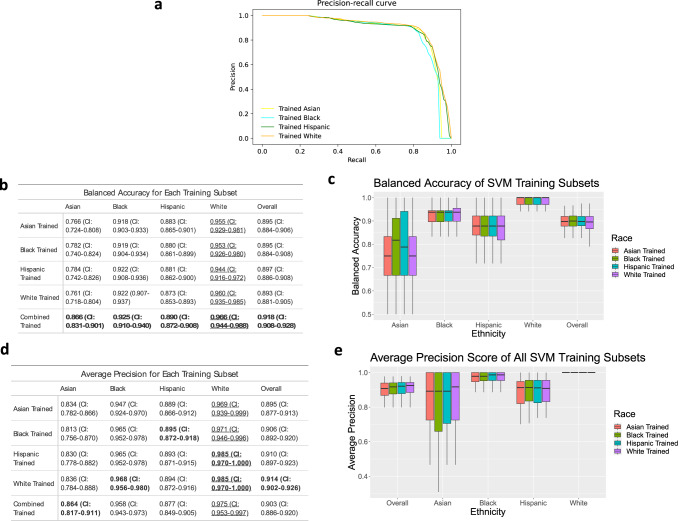
Ethnicity-specific training of the best overall model (SVM). Data was collected using 10 runs of 5-fold cross-validation. **a** Precision-recall curves for each model trained. **b**, **d** The balanced accuracy (**b**) and average precision (**d**) of each training as an average of the 50 runs. Bold means the highest performance among methods (columns), and underline means the highest performance among ethnicities (rows). **c**, **e** Boxplots showing the median, upper quartile and lower quartile of the balanced accuracy (**c**) and average precision (**e**). Outliers are excluded. Values for boxplots are available in Supplementary Tables 4 and 5.

SVM models were then trained on subsets of data that contained only one of the four ethnicities. The boxplot in Fig. 3c shows that the trends shown in Fig. 1c are maintained. The balanced accuracy and average precision (Fig. 3d, e) of the models are the best on white women, followed by Black, Hispanic, and Asian women.

Comparison of the tables in Figs. 1b, d and 3b, d show that in many cases, training the models on subsets of ethnicity decreases performance. When evaluating how a specifically trained model performs on its relative population of women (e.g., a model trained on Asian women predicting BV positivity for Asian women), all models have a worse balanced accuracy than when trained on the entire set of data. The average precisions show a similar trend for models trained individually on Asian and Hispanic subsets. These trends can be attributed to the decreased number of samples that are available to train the model. However, when assessing average precision (AP), models trained on the Black and white subsets have a higher average precision when predicting BV in their relative populations.

### Ethnicity-specific feature selection with model performance

**Fig. 4:**
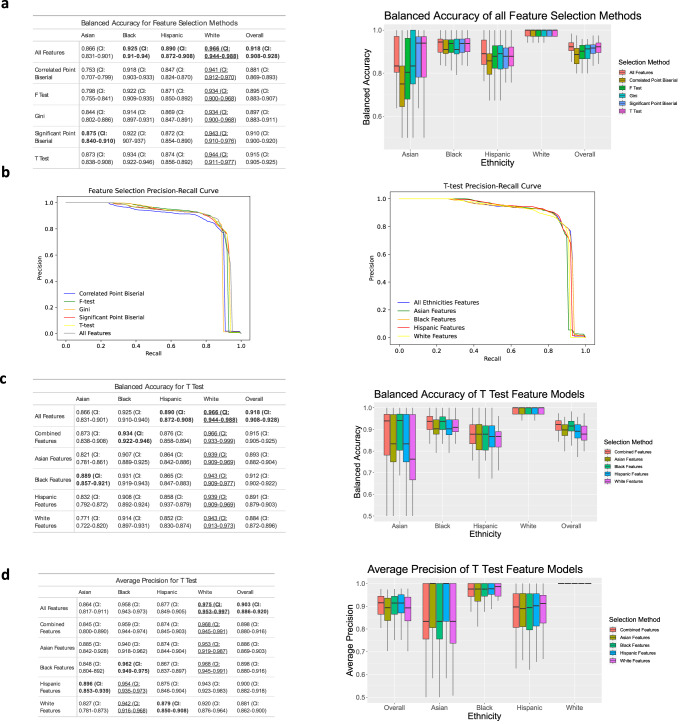
Selecting the most important features for each ethnicity run on the SVM model. **a** A table and plot showing the balanced accuracy for each feature selection method run on the SVM model. **b** Precision Recall Curves and corresponding adjusted precision values for each of the selection methods. **c** Balanced Accuracy table and plot. Results show features selected for each ethnicity using the T Test method tested against each ethnicity. **d** Average Precision table and plot for the same test run in **c**. Outliers are excluded. Values for boxplots are available in Supplementary Tables 7–9. Bold means the highest performance among methods (columns), and underline means the highest performance among ethnicities (rows).

To increase the model performance, feature selection methods were implemented. Feature selection identifies variables that are important for the model to predict BV and reduces variables that may not be beneficial. The SVM model has the highest balanced accuracy when all features are used to train the model; however, feature selection methods result in a similar accuracy (Fig. 4a). Within this study, ethnicity-specific feature selection does not significantly improve balanced accuracy for white, Hispanic, and Asian subsets; however, it does improve the balanced accuracy for the Black subset (from 0.925 to 0.931) using Black features. Based on the metric of balanced accuracy, the T-test feature selection method performs best on the overall test set (0.915), and is used for subsequent analysis (Fig. 4c, d).

In Fig. 4a, the white subset had the highest balanced accuracy using all features (0.966), and remains the highest, even when trained using different feature selection methods. Feature selection using Correlated Point Biserial performs the worst out of all the tests, with particularly low performance for the Asian subset (0.753). In the multiple runs of the SVM, the Asian subset displays the greatest variability, followed by the Hispanic subset.

In Fig. 4b, precision-recall curves show that the ethnicity-specific feature tests have similar average precision to training with all features. In the correlated Point Biserial, F-Test, and Gini precision-recall curves, the Black feature set has the highest average precision at 0.8523, 0.822, and 0.86 respectively.

In Fig. 4c, d, the balanced accuracy and average precision values are compared using features selected from different ethnicity groups via T-Test feature selection. The high balanced accuracy of Black features for all ethnicity groups indicates that features selected from the Black subset have a good representation of important features to diagnose BV. Interestingly, the Asian subset has the higher balanced accuracy when trained using the Black features (0.889) compared to using all features or features selected from all ethnicities. The Asian subset has the most variability with each feature set and the lowest accuracy when trained on white and Hispanic feature sets. The balanced accuracy of the white subset is the highest, even when using features selected using other ethnicity groups. In contrast, using the metric of average precision (Fig. 4d), the best performances are achieved by using the Hispanic feature set for the Asian subset, the Black feature set for Black and white, and the white feature set for the Hispanic subset.

**Fig. 5:**
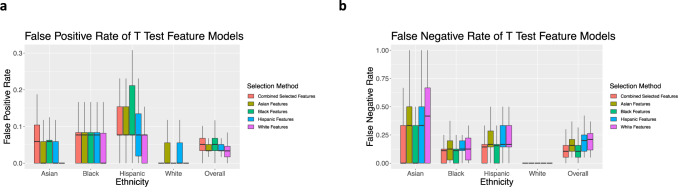
False positive and false negative rates for SVM models using T Test feature selection. **a** The false positive rate for the T Test feature selection method tested on each ethnicity. **b** The false negative rate for the T Test feature selection method tested on each ethnicity. Outliers are excluded. Values for boxplots are available in Supplementary Tables 10 and 11.

When evaluating the FPR of the feature selection, FPR is the highest overall when training with the combined selected features, which are selected using data from the entire dataset (Fig. 5a). Interestingly, the model using the selected features for white women has the lowest FPR for each ethnicity. Across all feature sets, the model performs the worst for Hispanic women.

When examining the FNR of the feature test experiments, the overall FNR is the highest when training the model with selected features for white women, followed by selected features for Hispanic, Asian, and Black women, respectively (Fig. 5b). When examining the FNR by ethnicity, the FNR is highest for Asian women, regardless of ethnicity-specific features.

### Feature selection reveals important microbes in BV patients of different ethnicities

**Fig. 6:**
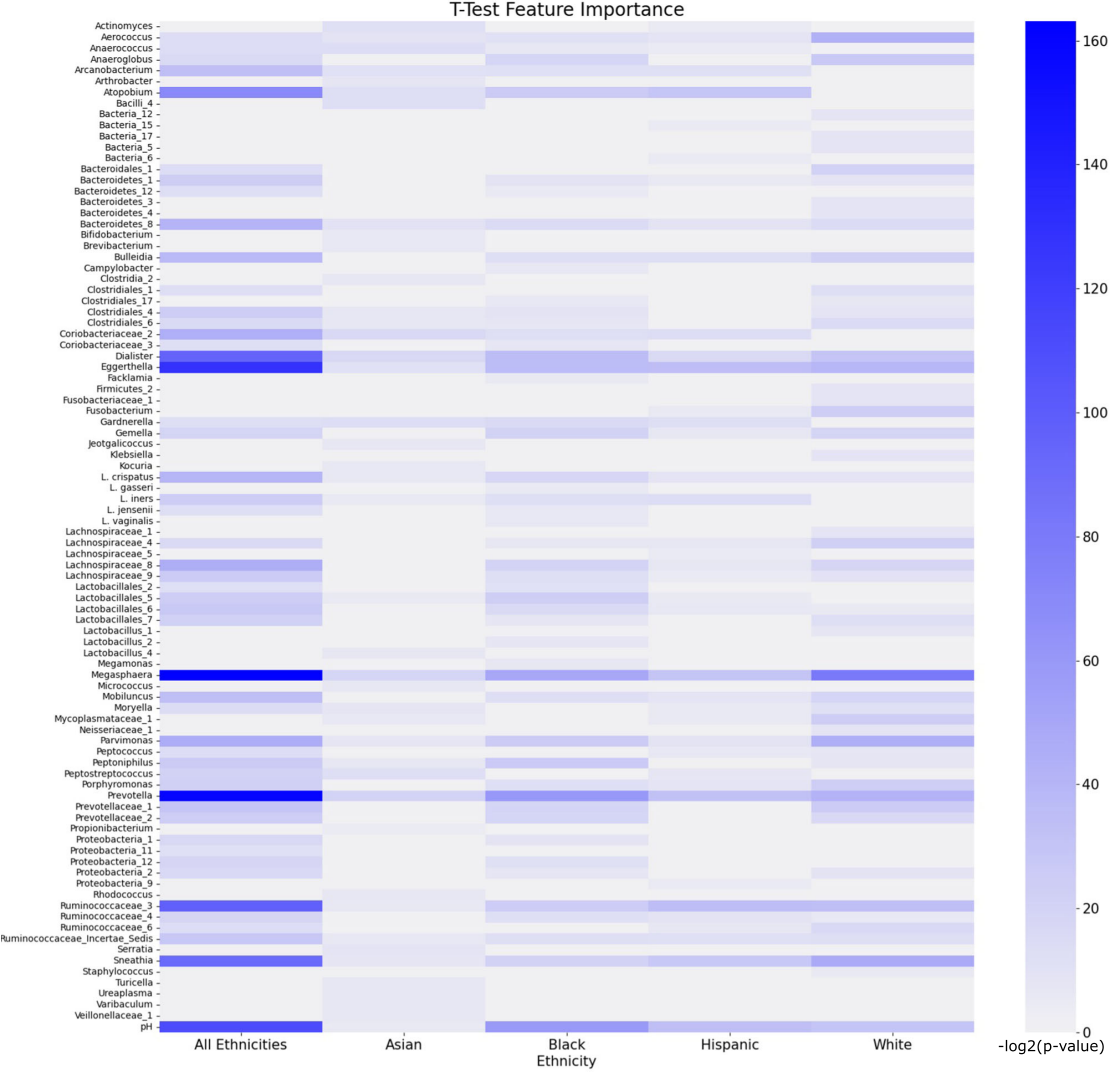
A heatmap showing the top ~50 features selected by the T Test method for the SVM model. The intensity of the heatmap shows higher significance. The corresponding *p*-values are log transformed. Features are ordered in alphabetical order. Values for the heatmap are available in Supplementary Table 12.

The heatmap in Fig. 6 shows the most important features returned when applying the T-Test to each group. *Atopobium, Dialister, Eggerthella, Megasphaera, Prevotella, Ruminococcaceae_3, Sneathia*, and pH show strong importance when using the T-Test on the “all ethnicities” group. *Prevotella, Megasphaera, Gardnerella, and Dialister* are shown to be the Asian feature set’s top features. Significant features derived from the Asian subset are not as strongly significant compared to the white, Black, and Hispanic-derived features (Supplementary Table 1). The Black feature set shows that *Megasphaera, Prevotella*, and pH show a strong significance (*p* = 1.03e-15 to 6.58e-19). To a lesser degree, *Atopobium, Eggerthella, Dialister, Parvimonas, and Peptnophilus* are shown to also have high significance to the Black feature set (*p* = 1.52e-8 to 1.5e-11). The Hispanic feature set is similar to the Black feature set with the top significant features being *Megasphaera, Prevotella*, pH, and *Atopobium*. *Sneathia* and *Ruminococcaceae_3* are shown to be more significant to the Hispanic feature set than to the Black feature set. The most significant features for the white subset are *Megasphaera, Sneathia, Parvimonas, Aerococcus, Prevotella*, and *Eggerthella* respectively. The Black and white feature sets have a greater number of significant features compared to the Hispanic and Asian feature sets. The most consistent significant features across all groups are *Megasphaera, Eggerthella, Prevotella, Dialister, Sneathia, Ruminococcaceae_3*, and pH.

## Discussion

The application of Fairness in AI allows for the evaluation of ML model performance across ethnicity^25^. Here, we show that several supervised learning models perform differently for ethnic groups by assessing commonly used metrics, such as balanced accuracy and average precision, as well as more clinically relevant metrics, such as FPR and FNR, in a cohort of women with asymptomatic BV. The results provide evidence that there is a discrepancy in model performance between ethnicities.

The selected models (RF, SVM, LR, MLP) vary in performance using the metrics of balanced accuracy and precision; however, the models generally perform most effectively on the white subset and the least effectively on the Asian subset (Fig. 1). Interestingly, Random Forest performs best for the Black subset in both balanced accuracy and precision. These findings highlight the fact that the diversity of microbial composition seen in Black women may be more accurately predicted using a tree-based model.

When using metrics of importance for clinical diagnosis, such as FPR, Hispanic and Black women are most affected, with Hispanic women having the highest FPR (Fig. 2a, b). False positive rates indicate a misdiagnosis of BV in women, which can result in unnecessary costs and incorrect treatment for patients, including over-prescription of antibiotics which can have negative effects^15,31,32^. In contrast, the FNR indicates an incorrect negative diagnosis, which can result in lack of necessary treatment, increasing the risk of associated adverse events (e.g., STDs, pelvic inflammatory disease, infertility, early labor). A failure to screen for BV in the asymptomatic state can have long-lasting effects on the quality of life. In the case of this work, Hispanic and Asian women have the highest FNR across models, highlighting the potential for inadequate treatment and increased health risk for these populations.

To begin to mitigate this discrepancy and create a fair machine learning classifier, we test whether ethnicity-specific feature sets improve accuracy. Our results show that this method does increase precision for Black and Asian subsets (Fig. 4d). In addition, these ethnicity-specific feature selections provide insight into the differences between BV presentation in women of different ethnicities. With respect to FPR and FNR, the findings of the ethnicity-specific feature selections were similar to those of models without feature selections.

The inequal performance of the models could be partially due to the imbalance of the dataset, which can make it appear that a model is performing better than it would in a clinical setting. BV-positive samples for the white and Asian populations were limited in this dataset. However, there is a consistent trend that the models performed the best on the white subset and the worst on the Asian subset. This suggests that there are other factors involved. One such factor may be the baseline complexity of the vaginal microbiome in Black, Asian, and Hispanic women^17,18^.

The variability in definition of a healthy vaginal microbiome could lead to inequal performance of general purpose, one-size-fits-all ML models. The different distribution of microbes provides domain-informed prior information for ML models that can account for differences in prediction accuracy. Vaginal CSTs begin to characterize the complexity of the vaginal microbiome, by defining dominant bacteria, ranging from I-V^19^. It is seen in this dataset that the majority of Black and Hispanic women belong to community group IV, which is the most complex, and has a high prevalence of *Prevotella, Dialister, Atopobium, Gardnerella, Megasphaera, Peptoniphilus, Sneathia, Eggerthella, Aerococcus, Finegoldia*, and *Mobiluncus*. Other community groups are dominated by *Lactobacillus* spp.^16^. This could explain why the models perform worse for Black and Hispanic women than they do for white women. The majority of women in the Asian subset did not categorize as community group IV; however, it had the most even distribution of patients across all community groups. This is supported by the heatmap in Fig. 6, which offers minimal evidence for the importance of a singular microbe to distinguish between BV positive and BV negative in Asian women. At the same time, the heatmap provides strong support for the importance of certain bacteria, such as *Megasphaera*, for the diagnosis of white women. Finally, we are able to see shared features that are important for each subgroup of women, which can inform fair molecular diagnostics^33^.

The ethnicity-specific feature selection provides evidence that information from one ethnic group can assist in the decision-making ability of a model on another ethnic group. Figure 3 shows that training the model using Black and Hispanic features improves model performance for Asian women, even greater than when the model is trained using features identified from the Asian subset. This phenomenon could be attributed to the fact that the variety of data in the subsets of Black/Hispanic women and Asian women are comparable. Using these feature selections, the model can leverage the more balanced nature of Black/Hispanic subsets and make better predictions on the unbalanced subset of Asian participants.

It is important to recognize that these results may not be generalizable due to the small sample size of the dataset. In addition, the small sample sizes of the Asian and white populations with a positive BV diagnosis may impact statistical validity when evaluating the model performance and selecting the most important features with respect to these racial populations. However, it is important to note that even with these limitations, our models identify discrepancies in accuracy across ethnicity groups.

Due to the increasing availability and use of 16S rRNA sequencing to characterize BV, the possibility of using ML models is important to consider, as they can be leveraged as powerful tools to determine the relationship between the vaginal microbiome composition and adverse outcomes^34–38^. Traditional diagnostic tools can be limited, and more information is needed to determine nuances based on ethnicity^17^. ML models developed on data from women of multiple ethnicities can help parse individual differences for more accurate diagnosis and better therapeutics.

Future work should include expanding studies focused on collecting vaginal microbiome data with respect to BV diagnosis to improve statistical validity of ML models for predicting BV. These studies should prioritize the collection of a balanced dataset in regard to BV-negative and BV-positive patients in each ethnicity. Furthermore, within this asymptomatic dataset, women previously diagnosed with BV could be indicated, which could provide added benefit to the ML model and further develop it to provide an optimal performance. Due to the importance of community group and the complexity of a patient’s vaginal microbiome, future studies should also focus on collecting data from women of different community state types. Lastly, future work should focus on improving ML and AI models to detect positive BV diagnosis with respect to differentiation in the vaginal microbiome across ethnicity, regardless of data imbalance.

## Methods

### Dataset

The dataset was originally reported by Ravel et al.^16^. The study was registered at clinicaltrials.gov under ID NCT00576797. The protocol was approved by the institutional review boards at Emory University School of Medicine, Grady Memorial Hospital, and the University of Maryland School of Medicine. Written informed consent was obtained by the authors of the original study.

### Preprocessing

Samples were taken from 394 asymptomatic women. 97 of these patients were categorized as positive for BV, based on Nugent score. In the preprocessing of the data, information about community group, ethnicity, and Nugent score was removed from the training and testing datasets. Ethnicity information was stored to be referenced later during the ethnicity-specific testing. 16S rRNA values were listed as a percentage of the total 16S rRNA sample, so those values were normalized by dividing by 100. pH values ranged on a scale from 1 to 14 and were normalized by dividing by 14.

### Multiple runs

Each experiment was run 10 times, with a different random seed defining the shuffle state, to gauge variance of performance.

### Supervised machine learning

Four supervised machine learning models were evaluated. Logistic regression (LR), support vector machine (SVM), random forest (RF), and Multi-layer Perceptron (MLP) models were implemented with the scikit-learn python library. LR fits a boundary curve to separate the data into two classes. SVM finds a hyperplane that maximizes the margin between two classes. These methods were implemented to test whether boundary-based models can perform fairly among different ethnicities. RF is a model that creates an ensemble of decision trees and was implemented to test how a decision-based model would classify each patient. MLP passes information along nodes and adjusts weights and biases for each node to optimize its classification. MLP was implemented to test how a neural network-based approach would perform fairly on the data.

### K-folds cross-validation

Five-fold stratified cross validation was used to prevent overfitting and to ensure that each ethnicity has at least two positive cases in the test folds. Data were stratified by a combination of ethnicity and diagnosis to ensure that each fold has every representation from each group with comparable distributions.

### Hyper parameter tuning

For each supervised machine learning model, hyper parameter tuning was performed by employing a grid search methodology from the scikit-learn python library. Nested cross validation with 4 folds and 2 repeats was used as the training subset of the cross validation scheme.

### Hyper parameters

For Logistic Regression, the following hyper-parameters were tested: solver (newton-cg, lbfgs, liblinear) and the inverse of regularization strength C (100, 10, 1.0, 0.1, 0.01).

For SVM, the following hyper-parameters were tested: kernel (polynomial, radial basis function, sigmoid) and the inverse regularization parameter C (10, 1.0, 0.1, 0.01).

For Random Forest, the following hyper-parameters were tested: number of estimators (10, 100, 1000) and maximum features (square root and logarithm to base 2 of the number of features).

For Multi-layer perceptron, the following hyper-parameters were tested: hidden layer size (3 hidden layers of 10,30, and 10 neurons and 1 hidden layer of 20 neurons), solver (stochastic gradient descent and Adam optimizer), regularization parameter alpha (0.0001, or .05), and learning rate (constant and adaptive).

### Metrics

The models were evaluated using the following metrics: balanced accuracy, average precision, false positive rate (FPR), and false negative rate (FNR). Balanced accuracy was chosen to better capture the practical performance of the models while using an unbalanced dataset. Average precision is an estimate of the area under the precision recall curve, similar to AUC which is the area under the ROC curve. The precision-recall curve is used instead of a receiver operator curve to better capture the performance of the models on an unbalanced dataset^39^. Previous studies with this dataset reveal particularly good AUC scores and accuracy, which is to be expected with a highly unbalanced dataset.

The precision-recall curve was generated using the true labels and predicted probabilities from every fold of every run to summarize the overall precision-recall performance for each model. Balanced accuracy and average precision were computed using the corresponding functions found in the sklearn.metrics package. FPR and FNR were calculated computed and coded using Equations below^39^.

Below are the equations for the metrics used to test the Supervised Machine Learning models:

1 $${Precision}=\frac{{TP}}{{TP}+{FP}}$$

2 $${Recall}=\frac{{TP}}{{TP}+{FN}}$$

3 $${Balanced}\,{Accuracy}=\frac{1}{2}\left(\frac{{TP}}{{TP}+{FN}}+\frac{{TN}}{{TN}+{FP}}\right)$$

4 $${FPR}=\frac{{FP}}{{FP}+{TN}}$$

5 $${FNR}=\frac{{FN}}{{FN}+{TP}}$$

where TP is the number of true positives, TN is the number of true negatives, FP is the number of false positives, and FN is the number of false negatives.

6 $${Average}\,{Precison}=\sum _{n}\left({R}_{n}-{R}_{n-1}\right){P}_{n}$$

where R denotes recall, and P denotes precision.

### Ethnicity specific testing

The performance of the models were tested against each other as previously stated. Once the model made a prediction, the stored ethnicity information was used to reference which ethnicity each predicted label and actual label belonged to. These subsets were then used as inputs for the metrics functions.

To see how training on data containing one ethnicity affects the performance and fairness of the model, an SVM model was trained on subsets that each contained only one ethnicity. Information on which ethnicity each datapoint belonged to was not given to the models.

#### Feature selection

To increase the performance and accuracy of the model, several feature selection methods were used to reduce the 251 features used to train the machine learning models. These sets of features were then used to achieve similar or higher accuracy with the machine learning models used. The feature selection methods used included the ANOVA F-test, two-sided T-Test, Point Biserial correlation, and the Gini impurity. The libraries used for these feature selection tests were the statistics and scikit learn packages in Python. Each feature test was performed with all ethnicities, then only the white subset, only Black, only Asian, and only Hispanic.

The ANOVA F-Test was used to select 50 features with the highest F-value. The function used calculates the ANOVA F-value between the feature and target variable using variance between groups and within the groups. The formula used to calculate this is defined as:

7 $$F=\frac{{SSB}/(k-1)}{{SSW}/(n-k)}$$

Where k is the number of groups, n is the total sample size, SSB is the variance between groups, and SSW is the sum of variance within each group. The two-tailed T-Test was used to compare the BV negative versus BV positive group’s rRNA data against each other. The two-tailed T-Test is used to compare the means of two independent groups against each other. The null hypothesis in a two-tailed T-Test is defined as the means of the two groups being equal while the alternative hypothesis is that they are not equal. The dataset was split up into samples that were BV negative and BV positive which then compared the mean of each feature against each other to find significant differences. A p-value <0.05 allows us to reject the null hypothesis that the mean between the two groups is the same, indicating there is a significant difference between the positive and negative groups for each feature. Thus, we use a p-value of less than 0.05 to select important features. The number of features selected were between 40 and 75 depending on the ethnicity group used. The formula for finding the t-value is defined as:

8 $$t=\frac{\left({\bar{x}}_{1}-{\bar{x}}_{2}\right)}{\sqrt{\frac{({{s}_{1}})^{2}}{{n}_{1}}+\frac{({{s}_{2}})^{2}}{{n}_{2}}}}$$

${\bar{{\rm{x}}}}_{1,2}$ being the mean of the two groups. ${{\rm{s}}}_{1,2}$ as the standard deviation of the two groups. ${{\rm{n}}}_{1,2}$ being the number of samples in the two groups. The p-value is then found through the *t*-value by calculating the cumulative distribution function. This defines probability distribution of the t-distribution by the area under the curve. The degrees of freedom are also needed to calculate the *p*-value. They are the number of variables used to find the *p*-value with a higher number being more precise. The formulas are defined as:

9 $${\rm{df}}={n}_{1}+{n}_{2}{{{-}}}2$$

10 $${p}=2* \left(1-{\rm{CDF}}\left(\left|t\right|,{\rm{df}}\right)\right)$$

where ${df}$ denotes the degrees of freedom and ${{\rm{n}}}_{1,2}$ being the number of samples in the group. The Point Biserial correlation test is used to compare categorical against continuous data. For our dataset was used to compare the categorical BV negative or positive classification against the continuous rRNA bacterial data. Each feature has a *p*-value and correlation value associated with it which was then restricted by an alpha of 0.2 and further restricted by only correlation values >0.5 showing a strong correlation. The purpose of the alpha value is to indicate the level of confidence of a *p*-value being significant. An alpha of 0.2 was chosen because the Point Biserial test tends to return higher *p*-values. This formula is defined as:

11 $${{r}}_{{pb}}=\frac{\left({M}_{1}-{M}_{0}\right)}{{\rm{s}}}\,\sqrt{{pq}}$$

where M1 is the mean of the continuous variable for the categorical variable with a value of 1; M0 is the mean of the continuous variable for the categorical variable with a value of 0; s denotes the standard deviation of the continuous variable; p is the proportion of samples with a value of 1 to the sample set; and q is the proportion of samples with a value of 0 to the sample set.

Two feature sets were made from the Point Biserial test. One feature set included only the features that were statistically significant using a *p*-value of <0.2 which returned 60–100 significant features depending on the ethnicity set used. The second feature set included features that were restricted by a *p*-value < 0.2 and greater than a correlation value of 0.5. This second feature set contained 8–15 features depending on the ethnicity set used.

Features were also selected using Gini impurity. Gini impurity defines the impurity of the nodes which will return a binary split at a node. It will calculate the probability of misclassifying a randomly chosen data point. The Gini impurity model fitted a Random Forest model with the dataset and took the Gini scores for each feature based on the largest reduction of Gini impurity when splitting nodes. The higher the reduction of Gini value, the impurity after the split, the more important the feature is used in predicting the target variable. The Gini impurity value varies between 0 and 1. Using Gini, the total number of features were reduced to 3–10 features when using the ethnicity-specific sets and 20 features when using all ethnicities. The formula is defined as:

12 $${Gini}=1-\sum {{p}_{i}}^{2}$$

where ${{\rm{p}}}_{{\rm{i}}}$ is the proportion of each class in the node. The five sets of selected features from each of the five ethnicities were used to train a model using four supervised machine learning algorithms (LR, MLP, RF, SVM) with the full dataset using our nested cross-validation schemed as previously described. All features were selected using the training sets only, and they were applied to the test sets after being selected for testing. Five-fold stratified cross validation was used for each model to gather including means and confidence intervals.

### Supplementary information

Supplementary Tables

## Data Availability

The bacterial 16S rRNA gene sequences datasets analyzed during the current study are available in the National Center for Biotechnology Information Short Read Archive (SRA022855). Data on ethnicity [https://www.pnas.org/doi/full/10.1073/pnas.1002611107].

## Code availability

The code used to run experiments and generate results is available on GitHub at [https://github.com/ikenn04/Fair-AI-Asymptomatic-BV/tree/main]. A README file explaining the details of the repository and how to run the code is included.
