# Supplementary material for: Ethnic disparity in diagnosing asymptomatic bacterial vaginosis using machine learning
Source: NPJ Digit Med. 2023 Nov 17;6:211. doi: 10.1038/s41746-023-00953-1 (PMC10656445; doi:10.1038/s41746-023-00953-1)
Supplement: Supplementary file 1 — Supplementary Tables [file 41746_2023_953_MOESM1_ESM.pdf]

	Model			
Plot Component	Logistic Regression	MLP Classifier	Random Forest	SVM
Asian Min	0.5	0.5	0.470588	0.5
Asian LQ	0.75	0.76348	0.5	0.803922
Asian Mean	0.803922	0.833333	0.666667	0.833333
Asian UQ	0.970588	1	0.75	0.970588
Asian Max	1	1	0.833333	1
Black Min	0.798077	0.5	0.833333	0.791667
Black LQ	0.895833	0.888889	0.916667	0.896635
Black Mean	0.927083	0.9375	0.944444	0.944444
Black UQ	0.944444	0.954861	0.961538	0.958333
Black Max	1	1	1	1
Hispanic Min	0.78022	0.5	0.711538	0.762821
Hispanic LQ	0.839744	0.834936	0.822344	0.839744
Hispanic Mean	0.89011	0.884615	0.867674	0.89011
Hispanic UQ	0.928571	0.916667	0.910027	0.953297
Hispanic Max	1	1	1	1
White Min	0.472222	0.472222	0.472222	0.720588
White LQ	0.970997	0.970588	0.75	0.972222
White Mean	1	1	0.986111	1
White UQ	1	1	1	1
White Max	1	1	1	1
Overall Min	0.833051	0.5	0.783051	0.808207
Overall LQ	0.881105	0.891525	0.866999	0.904174
Overall Mean	0.914035	0.912719	0.884657	0.922368
Overall UQ	0.933051	0.932793	0.899894	0.941102
Overall Max	0.983333	0.966525	0.966525	0.983333

Supplementary Table 1. Values for boxplot components in Figure 1c.

Plot Component	Model			
	Logistic Regression	MLP Classifier	Random Forest	SVM
Asian Min	0.752801	0.74563	0.765778	0.731379
Asian LQ	0.869687	0.887809	0.8766	0.876432
Asian Mean	0.909871	0.919358	0.921147	0.920598
Asian UQ	0.945529	0.947088	0.94457	0.942273
Asian Max	0.977488	0.990847	0.981277	0.981277
Black Min	0.444444	0.571429	0.297258	0.361111
Black LQ	0.75	0.809524	0.679825	0.751389
Black Mean	0.833333	1	0.833333	0.916667
Black UQ	1	1	1	1
Black Max	1	1	1	1
Hispanic Min	0.729302	0.760268	0.835615	0.70506
Hispanic LQ	0.950891	0.946781	0.962529	0.94675
Hispanic Mean	0.976543	0.986111	0.988889	0.975
Hispanic UQ	1	1	1	0.997222
Hispanic Max	1	1	1	1
White Min	0.680159	0.720501	0.640512	0.621032
White LQ	0.803033	0.839989	0.812571	0.831207
White Mean	0.910256	0.914683	0.91182	0.900992
White UQ	0.965774	0.969246	0.961735	0.948323
White Max	1	1	1	1
Overall Min	0.583333	0.305556	0.392857	0.583333
Overall LQ	1	0.833333	0.666667	1
Overall Mean	1	1	1	1
Overall UQ	1	1	1	1
Overall Max	1	1	1	1

Supplementary Table 2. Values for boxplot components in Figure 1e.

Plot Component	Model			
	Logistic Regression	MLP Classifier	Random Forest	SVM
Asian Min	0	0	0	0
Asian LQ	0	0	0	0
Asian Mean	0	0	0	0.058824
Asian UQ	0.058824	0	0	0.061581
Asian Max	0.125	0.117647	0.058824	0.125
Black Min	0	0	0	0
Black LQ	0	0	0	0
Black Mean	0.076923	0.076923	0.076923	0.076923
Black UQ	0.083333	0.083333	0.083333	0.083333
Black Max	0.166667	0.307692	0.166667	0.230769
Hispanic Min	0	0	0	0
Hispanic LQ	0	0.019231	0	0.076923
Hispanic Mean	0.076923	0.076923	0.076923	0.076923
Hispanic UQ	0.153846	0.153846	0.134615	0.153846
Hispanic Max	0.230769	0.307692	0.230769	0.307692
White Min	0	0	0	0
White LQ	0	0	0	0
White Mean	0	0	0	0
White UQ	0.055556	0	0.041667	0.055556
White Max	0.117647	0.058824	0.117647	0.117647

Supplementary Table 3. Values for boxplot components in Figure 2a.

Plot Component	Model<br>Logistic Regression	MLP Classifier	Random Forest	SVM
Asian Min	0	0	0.333333	0
Asian LQ	0	0	0.5	0
Asian Mean	0.333333	0.333333	0.666667	0.333333
Asian UQ	0.5	0.458333	1	0.333333
Asian Max	1	1	1	1
Black Min	0	0	0	0
Black LQ	0.111111	0	0	0
Black Mean	0.125	0.125	0	0.111111
Black UQ	0.125	0.125	0.125	0.125
Black Max	0.333333	1	0.25	0.25
Hispanic Min	0	0	0	0
Hispanic LQ	0	0.142857	0.142857	0
Hispanic Mean	0.142857	0.166667	0.166667	0.142857
Hispanic UQ	0.166667	0.166667	0.285714	0.166667
Hispanic Max	0.333333	1	0.5	0.333333
White Min	0	0	0	0
White LQ	0	0	0	0
White Mean	0	0	0	0
White UQ	0	0	0.5	0
White Max	1	1	1	0.5

Supplementary Table 4. Values for boxplot components in Figure 2b.

Plot Component	Training Subset Asian	Black	Hispanic	White
Asian Min	0.5	0.5	0.5	0.5
Asian LQ	0.666667	0.666667	0.666667	0.666667
Asian Mean	0.75	0.817708	0.788297	0.75
Asian UQ	0.833333	0.911765	0.941176	0.833333
Asian Max	1	1	1	1
Black Min	0.798077	0.759615	0.833333	0.798077
Black LQ	0.896635	0.896635	0.899038	0.899038
Black Mean	0.9375	0.9375	0.9375	0.9375
Black UQ	0.944444	0.944444	0.944444	0.954861
Black Max	1	1	1	1
Hispanic Min	0.717949	0.717949	0.717949	0.717949
Hispanic LQ	0.839744	0.834936	0.834936	0.818681
Hispanic Mean	0.878205	0.878205	0.878205	0.878205
Hispanic UQ	0.921474	0.921474	0.921474	0.921474
Hispanic Max	1	1	1	1
White Min	0.720588	0.661765	0.720588	0.720588
White LQ	0.970588	0.970588	0.970588	0.970588
White Mean	1	1	1	1
White UQ	1	1	1	1
White Max	1	1	1	1
Overall Min	0.790366	0.790366	0.790366	0.790366
Overall LQ	0.877858	0.877858	0.879315	0.866682
Overall Mean	0.897814	0.899788	0.897814	0.895841
Overall UQ	0.919815	0.921722	0.919815	0.919815
Overall Max	0.96521	0.966667	0.975	0.975

Supplementary Table 5. Values for boxplot components in Figure 3c.

	Training Subset			
Plot Component	Asian	Black	Hispanic	White
Asian Min	0.682479	0.777831	0.798514	0.798514
Asian LQ	0.868019	0.876218	0.878326	0.884718
Asian Mean	0.90768	0.917367	0.920906	0.924356
Asian UQ	0.939086	0.939983	0.942432	0.942764
Asian Max	0.976849	0.976849	0.979284	0.976849
Black Min	0.466667	0.309524	0.466667	0.466667
Black LQ	0.72549	0.659722	0.705556	0.72549
Black Mean	0.891667	0.891667	0.891667	0.916667
Black UQ	1	1	1	1
Black Max	1	1	1	1
Hispanic Min	0.633667	0.771379	0.782341	0.801524
Hispanic LQ	0.946781	0.9521	0.953526	0.953526
Hispanic Mean	0.978171	0.978171	0.986111	0.986111
Hispanic UQ	1	1	1	1
Hispanic Max	1	1	1	1
White Min	0.70102	0.706515	0.7375	0.7375
White LQ	0.819898	0.835265	0.824779	0.831611
White Mean	0.912698	0.913178	0.911026	0.908447
White UQ	0.948323	0.95496	0.95496	0.95496
White Max	1	1	1	1
Overall Min	0.45	0.583333	0.75	0.75
Overall LQ	1	1	1	1
Overall Mean	1	1	1	1
Overall UQ	1	1	1	1
Overall Max	1	1	1	1

Supplementary Table 6. Values for boxplot components in Figure 3e.

Plot Component	Feature Selection Method: All Features	Correlated Point Biserial	F Test	Gini	Significant Point Biserial	T Test
Asian Min	0.500	0.500	0.500	0.500	0.500	0.500
Asian LQ	0.804	0.645	0.680	0.750	0.781	0.781
Asian Mean	0.833	0.750	0.804	0.833	0.939	0.939
Asian UQ	0.971	0.833	0.962	1.000	0.969	0.970
Asian Max	1.000	1.000	1.000	1.000	1.000	1.000
Black Min	0.792	0.833	0.813	0.778	0.792	0.833
Black LQ	0.897	0.891	0.896	0.889	0.896	0.899
Black Mean	0.944	0.910	0.938	0.910	0.938	0.938
Black UQ	0.958	0.955	0.955	0.958	0.958	0.961
Black Max	1.000	1.000	1.000	1.000	1.000	1.000
Hispanic Min	0.763	0.673	0.673	0.673	0.756	0.756
Hispanic LQ	0.840	0.795	0.822	0.819	0.822	0.833
Hispanic Mean	0.890	0.857	0.878	0.890	0.878	0.878
Hispanic UQ	0.953	0.890	0.927	0.929	0.917	0.921
Hispanic Max	1.000	1.000	1.000	1.000	1.000	1.000
White Min	0.721	0.721	0.472	0.472	0.472	0.472
White LQ	0.972	0.971	0.971	0.971	0.971	0.971
White Mean	1.000	1.000	1.000	1.000	1.000	1.000
White UQ	1.000	1.000	1.000	1.000	1.000	1.000
White Max	1.000	1.000	1.000	1.000	1.000	1.000
Overall Min	0.808	0.799	0.799	0.733	0.825	0.825
Overall LQ	0.904	0.844	0.867	0.867	0.891	0.896
Overall Mean	0.922	0.886	0.902	0.913	0.916	0.922
Overall UQ	0.941	0.915	0.929	0.931	0.932	0.940
Overall Max	0.983	0.975	0.958	0.958	0.975	0.975

Supplementary Table 7. Values for boxplot components in Figure 4a.

Plot Component	Feature Set				
	Asian Features	Black Features	Hispanic Features	White Features	All Ethnicities
Asian Min	0.500	0.500	0.500	0.500	0.500
Asian LQ	0.750	0.804	0.750	0.667	0.781
Asian Median	0.833	0.941	0.833	0.762	0.939
Asian UQ	0.969	0.971	0.970	0.969	0.970
Asian Max	1.000	1.000	1.000	1.000	1.000
Black Min	0.760	0.833	0.771	0.792	0.833
Black LQ	0.878	0.899	0.875	0.889	0.899
Black Median	0.903	0.938	0.903	0.910	0.938
Black UQ	0.944	0.958	0.944	0.944	0.961
Black Max	1.000	1.000	1.000	1.000	1.000
Hispanic Min	0.673	0.756	0.673	0.673	0.756
Hispanic LQ	0.806	0.804	0.819	0.819	0.833
Hispanic Median	0.878	0.878	0.868	0.868	0.878
Hispanic UQ	0.923	0.917	0.917	0.890	0.921
Hispanic Max	1.000	1.000	1.000	1.000	1.000
White Min	0.721	0.472	0.721	0.721	0.472
White LQ	0.971	0.971	0.971	0.971	0.971
White Median	1.000	1.000	1.000	1.000	1.000
White UQ	1.000	1.000	1.000	1.000	1.000
White Max	1.000	1.000	1.000	1.000	1.000
Overall Min	0.799	0.825	0.773	0.799	0.825
Overall LQ	0.872	0.893	0.860	0.850	0.896
Overall Median	0.898	0.916	0.892	0.879	0.922
Overall UQ	0.922	0.938	0.922	0.916	0.940
Overall Max	0.958	0.983	0.958	0.975	0.975

Supplementary Table 8. Values for boxplot components in Figure 4c.

Plot Component	Feature Set				
	Asian Features	Black Features	Hispanic Features	White Features	All Ethnicities
Asian Min	0.703	0.733	0.726	0.702	0.732
Asian LQ	0.837	0.864	0.870	0.836	0.864
Asian Median	0.894	0.914	0.914	0.893	0.915
Asian UQ	0.936	0.944	0.951	0.940	0.944
Asian Max	0.973	0.976	0.977	0.977	0.976
Black Min	0.514	0.500	0.294	0.507	0.500
Black LQ	0.806	0.756	0.833	0.738	0.756
Black Median	1.000	0.833	1.000	0.833	0.833
Black UQ	1.000	1.000	1.000	1.000	1.000
Black Max	1.000	1.000	1.000	1.000	1.000
Hispanic Min	0.711	0.823	0.731	0.739	0.771
Hispanic LQ	0.922	0.944	0.952	0.945	0.943
Hispanic Median	0.976	0.975	0.977	0.986	0.976
Hispanic UQ	1.000	1.000	1.000	1.000	1.000
Hispanic Max	1.000	1.000	1.000	1.000	1.000
White Min	0.588	0.621	0.655	0.590	0.627
White LQ	0.810	0.796	0.813	0.826	0.807
White Median	0.889	0.894	0.902	0.912	0.897
White UQ	0.956	0.955	0.955	0.948	0.955
White Max	1.000	1.000	1.000	1.000	1.000
Overall Min	0.500	0.583	0.583	0.583	0.583
Overall LQ	1.000	1.000	1.000	1.000	1.000
Overall Median	1.000	1.000	1.000	1.000	1.000
Overall UQ	1.000	1.000	1.000	1.000	1.000
Overall Max	1.000	1.000	1.000	1.000	1.000

Supplementary Table 9. Values for boxplot components in Figure 4d.

Plot Component	Feature Set Asian Features	Black Features	Hispanic Features	White Features	All Ethnicities
Asian Min	0	0	0	0	0
Asian LQ	0	0	0	0	0
Asian Median	0	0.058824	0	0	0.058824
Asian UQ	0.058824	0.0625	0.058824	0	0.10386
Asian Max	0.117647	0.1875	0.117647	0.117647	0.1875
Black Min	0	0	0	0	0
Black LQ	0	0	0	0	0
Black Median	0.076923	0.076923	0.076923	0	0.076923
Black UQ	0.083333	0.083333	0.083333	0.081731	0.083333
Black Max	0.230769	0.166667	0.166667	0.166667	0.166667
Hispanic Min	0	0	0	0	0
Hispanic LQ	0.076923	0.076923	0.019231	0	0.076923
Hispanic Median	0.076923	0.076923	0.076923	0.076923	0.076923
Hispanic UQ	0.153846	0.211538	0.134615	0.076923	0.153846
Hispanic Max	0.230769	0.307692	0.230769	0.230769	0.307692
White Min	0	0	0	0	0
White LQ	0	0	0	0	0
White Median	0	0	0	0	0
White UQ	0.055556	0	0.055556	0	0
White Max	0.117647	0.058824	0.117647	0.058824	0.058824
Overall Min	0	0	0	0	0
Overall LQ	0.033333	0.033898	0.033333	0.016949	0.033898
Overall Median	0.033898	0.050847	0.033898	0.033333	0.050847
Overall UQ	0.050847	0.067797	0.050847	0.045975	0.067797
Overall Max	0.1	0.116667	0.084746	0.083333	0.101695

Supplementary Table 10. Values for boxplot components in Figure 5a.

	Feature Set				
Plot Component	Asian Features	Black Features	Hispanic Features	White Features	All Ethnicities
Asian Min	0	0	0	0	0
Asian LQ	0	0	0	0	0
Asian Median	0.333333	0	0.333333	0.416667	0
Asian UQ	0.5	0.333333	0.5	0.666667	0.333333
Asian Max	1	1	1	1	1
Black Min	0	0	0	0	0
Black LQ	0.027778	0	0.111111	0.027778	0
Black Median	0.125	0.111111	0.125	0.125	0.111111
Black UQ	0.197917	0.125	0.197917	0.222222	0.125
Black Max	0.375	0.25	0.375	0.333333	0.25
Hispanic Min	0	0	0	0	0
Hispanic LQ	0.142857	0	0.142857	0.142857	0
Hispanic Median	0.166667	0.154762	0.166667	0.166667	0.142857
Hispanic UQ	0.285714	0.166667	0.333333	0.333333	0.166667
Hispanic Max	0.5	0.333333	0.5	0.5	0.333333
White Min	0	0	0	0	0
White LQ	0	0	0	0	0
White Median	0	0	0	0	0
White UQ	0	0	0	0	0
White Max	0.5	1	0.5	0.5	1
Overall Min	0.05	0	0.05	0.05	0
Overall LQ	0.105263	0.052632	0.105263	0.116447	0.052632
Overall Median	0.157895	0.105263	0.2	0.210526	0.105263
Overall UQ	0.210526	0.157895	0.25	0.263158	0.157895
Overall Max	0.368421	0.315789	0.421053	0.368421	0.3

Supplementary Table 11. Values for boxplot components in Figure 5b.

Features	Ethnicity Subset				
	All Ethnicities	Asian	Black	Hispanic	White
Actinomyces	0	0.000353	0	0.03907	0
Aerococcus	6.27E-05	0.001521	0.000777	0.003125	1.10E-13
Anaerococcus	9.10E-05	8.03E-05	0.011694	0.044381	0
Anaeroglobus	1.92E-05	0	1.79E-06	0	6.03E-09
Arcanobacterium	7.81E-11	0.00084	0.000468	0.000358	0
Arthrobacter	0	0.006431	0	0	0
Atopobium	6.98E-22	0.000143	1.52E-08	1.74E-09	0
Bacilli_4	0	0.00022	0	0	0
Bacteria_12	0	0	0	0	0.002733
Bacteria_15	0	0	0	0.042124	0
Bacteria_17	0	0	0	0	0.002733
Bacteria_5	0	0	0	0	0.002733
Bacteria_6	0	0	0	0.042124	0
Bacteroidales_1	9.33E-05	0	0	0	6.48E-07
Bacteroidetes_1	9.83E-08	0	0.001347	0.027583	0.002733
Bacteroidetes_12	0.000198	0	0.024152	0	0
Bacteroidetes_3	0	0	0	0	0.002733
Bacteroidetes_4	0	0	0	0	0.002733
Bacteroidetes_8	5.40E-13	0.001066	3.53E-05	0.001632	3.78E-05
Bifidobacterium	0	0.018501	0	0	0
Brevibacterium	0	0.017405	0	0	0
Bulleidia	5.22E-12	0	0.000186	0.000228	2.89E-07
Campylobacter	0	0	0.0142	0	0
Clostridia_2	0	0.010756	0	0	0
Clostridiales_1	0.000336	0	0	0	0.000296
Clostridiales_17	0	0	0.017747	0	0.010419
Clostridiales_4	6.96E-08	0.010756	0.00193	0	0.002733
Clostridiales_6	2.38E-05	0.010756	0.005041	0	3.77E-05
Coriobacteriaceae_2	5.02E-14	1.43E-05	0.000166	7.97E-05	0
Coriobacteriaceae_3	0.000161	0	0.003298	0	0
Dialister	2.41E-29	8.02E-06	1.88E-11	1.88E-05	1.34E-09
Eggerthella	2.25E-39	0.000722	1.51E-11	3.32E-11	1.66E-12
Facklamia	0	0	0.018799	0	0
Firmicutes_2	0	0	0	0	0.002733
Fusobacteriaceae_1	0	0	0	0	0.002733
Fusobacterium	0	0	0	0.021061	6.87E-08
Gardnerella	0.000128	8.50E-05	2.38E-05	0.000338	0
Gemella	1.72E-06	0	5.81E-07	0.007856	1.33E-06
Jeotgalicoccus	0	0.010756	0	0	0
Klebsiella	0	0	0	0	0.002733
Kocuria	0	0.015318	0	0	0

L. crispatus	1.17E-12	0.017383	5.54E-06	0.003027	0.002555
L. gasseri	0	0	0.024631	0	0
L. iners	5.22E-08	0.024108	0.000112	9.35E-05	0
L. jensenii	0.000274	0	0.013299	0	0
L. vaginalis	0	0	0.014542	0	0
Lachnospiraceae_1	0	0	0	0	0.002733
Lachnospiraceae_4	2.07E-05	0	0.008915	0.008436	2.76E-07
Lachnospiraceae_5	0	0	0	0.042124	0
Lachnospiraceae_8	2.97E-14	0	7.86E-07	0.008839	3.67E-06
Lachnospiraceae_9	2.00E-08	0	0.00018	0.042124	0.000924
Lactobacillales_2	0.000322	0	0.000809	0	0
Lactobacillales_5	4.11E-08	0.022036	9.07E-08	0.022993	0
Lactobacillales_6	7.55E-09	0	2.21E-05	0.017793	0.021364
Lactobacillales_7	3.24E-07	0	0.007222	0	0.000165
Lactobacillus_1	0	0	0	0	0.005087
Lactobacillus_2	0	0	0.003737	0	0
Lactobacillus_4	0	0.010925	0	0	0
Megamonas	0	0	0.00809	0	0
Megasphaera	7.83E-50	2.34E-06	1.04E-15	1.50E-09	3.79E-25
Micrococcus	0	0.010756	0	0	0
Mobiluncus	8.33E-11	0	0.000299	0.002732	1.98E-06
Moryella	3.99E-05	0.004145	0	0.027258	0.000425
Mycoplasmataceae_1	0	0.012766	0	0.020398	7.39E-08
Neisseriaceae_1	0	0	0	0	0.002733
Parvimonas	1.60E-14	0.004418	1.42E-08	0.001895	4.71E-14
Peptococcus	8.77E-05	0	0	0.01563	0.006914
Peptoniphilus	1.64E-08	0.010652	1.31E-08	0	0.005185
Peptostreptococcus	5.28E-07	0.000111	0	0.005516	0
pH	3.12E-34	0.01998	9.61E-19	7.38E-11	8.59E-10
Porphyromonas	2.48E-07	0	0.000308	0.003012	4.12E-08
Prevotella	1.05E-48	1.06E-06	6.58E-19	2.03E-10	2.82E-13
Prevotellaceae_1	1.40E-09	0	1.31E-06	0	1.66E-08
Prevotellaceae_2	3.32E-08	0	1.85E-06	0	1.41E-05
Propionibacterium	0	0.047488	0	0	0
Proteobacteria_1	7.67E-06	0	0.001501	0	0
Proteobacteria_11	0.000409	0	0	0	0
Proteobacteria_12	3.05E-06	0	0.000386	0	0
Proteobacteria_2	1.04E-05	0	0.003365	0	0.001741
Proteobacteria_9	0	0	0	0.042124	0
Rhodococcus	0	0.010756	0	0	0
Ruminococcaceae_3	4.71E-30	0.013541	2.86E-08	3.85E-11	1.02E-10
Ruminococcaceae_4	3.72E-06	0	0.000333	0.001661	0.013418
Ruminococcaceae_6	7.35E-05	0	0	0.011844	1.23E-05
Ruminococcaceae_Incertae_Sedis	5.62E-09	0.012454	0.000117	0.000378	0.000127

Serratia	0	0.002638	0	0	0
Sneathia	4.39E-28	0.005018	5.16E-07	5.63E-09	3.27E-15
Staphylococcus	0	0	0	0	0.022598
Turicella	0	0.010756	0	0	0
Ureaplasma	0	0.008191	0	0	0
Varibaculum	0	0.008082	0	0	0
Veillonellaceae_1	0	0.010756	0	0	0

Supplementary Table 12. P values from the T-test feature selection method. Each column shows the subset that feature selection was performed on.
